# The Impact of Vehicle Occlusivity on Skin Delivery and Activity of a Janus Kinase Inhibitor: Comparison of Oil-Based Formulations

**DOI:** 10.3390/pharmaceutics18010008

**Published:** 2025-12-20

**Authors:** Paulo Sarango-Granda, Roya Mohammadi-Meyabadi, Antonio J. Braza, Lilian Sosa, Joaquim Suñer-Carbó, Mireia Mallandrich, Ana Cristina Calpena

**Affiliations:** 1 Departamento de Química, Facultad de Ciencias Exactas y Naturales, Universidad Técnica Particular de Loja (UTPL), Paris SN y Praga, Loja 1101607, Ecuador; pcsarango@utpl.edu.ec; 2 Departament de Farmàcia i Tecnologia Farmacèutica, i Fisicoquímica, Facultat de Farmàcia i Ciències de l’Alimentació, Universitat de Barcelona (UB), Av. Joan XXIII, 27-31, 08028 Barcelona, Spain; rmohammo31@alumnes.ub.edu (R.M.-M.); braza@ub.edu (A.J.B.); jsuner@ub.edu (J.S.-C.); 3 Instituto de Investigaciones en Microbiología (IIM), Facultad de Ciencias, Universidad Nacional Autónoma de Honduras (UNAH), Tegucigalpa 11101, Honduras; lilian.sosa@unah.edu.hn; 4 Centro Experimental en Biociencia (CENBIO), Facultad de Ciencias Químicas y Farmacia, Universidad Nacional Autónoma de Honduras (UNAH), Tegucigalpa 11101, Honduras; 5 Institut de Nanociència i Nanotecnologia, Universitat de Barcelona (UB), Av. Diagonal 645, 08028 Barcelona, Spain

**Keywords:** JAK1/JAK 2 inhibitor, topical delivery, occlusivity, lipid formulations, topical excipients, petrolatum, medium-chain triglycerides (MCT)

## Abstract

**Background/Objectives:** Baricitinib, a selective JAK1/JAK2 inhibitor, shows therapeutic potential in psoriasis; however, its oral use is associated with systemic adverse effects, encouraging the development of topical formulations. This study aimed at evaluating the influence of petrolatum type on the stability, biopharmaceutical performance, and therapeutic activity of lipid-based formulations containing Baricitinib. **Methods**: Formulations were prepared with Labrafac^®^ Lipophile WL 1349 (L) and either liquid (LLV) or solid (LSV) petrolatum at 30% and 60% *w*/*w*. Stability, rheology, spreadability, in vitro release, ex vivo permeation, and skin retention were evaluated, along with the safety and efficacy in HET-CAM and imiquimod-induced psoriasis murine models. **Results**: Only 30% petrolatum formulations remained stable for 60 days. LLV exhibited Newtonian flow, higher spreadability, sustained release (83.7% at 50 h), and superior skin retention (94 µg/g of skin/cm^2^), whereas LSV showed pseudoplastic behavior, lower spreadability, and reduced release (47.4% at 50 h). Both formulations were non-irritant and improved stratum corneum hydration while reducing transepidermal water loss. In vivo, both reduced erythema, epidermal thickening, edema, and histological alterations, confirming anti-inflammatory efficacy. **Conclusions**: These results demonstrate that the vehicle occlusivity decisively modulates baricitinib’s release and activity. LLV formulation favored drug retention and enhanced permeation at 24 h. Overall, excipient selection is important in designing safe and effective topical JAK inhibitor formulations.

## 1. Introduction

Psoriasis is a chronic, immune-mediated inflammatory disease that affects approximately 2–3% of the world’s population. It is a relapsing skin disorder that has a significant impact on the patients’ quality of life due to its visible, persistent, and clinically difficult-to-control nature. The pathophysiology of psoriasis is characterized by hyperproliferation of keratinocytes, infiltration of T lymphocytes and other immune cells, as well as the overexpression of proinflammatory mediators, resulting in thickened, scaly, erythematous plaques [1,2]. Psoriasis is also a risk factor for multiple comorbidities, including metabolic syndrome [3], cardiovascular disease [4], depression, and anxiety [5], accentuating its clinical and social relevance. In addition to immune dysregulation, psoriasis is characterized by a marked impairment of the epidermal barrier, resulting in increased transepidermal water loss and altered stratum corneum hydration.

Available therapeutic options include topical, systemic, and biological treatments. Topical agents, such as corticosteroids, retinoids, and vitamin D analogs, constitute the first-line treatment for mild-to-moderate forms [6]. However, their effectiveness may be limited, and long-term use is associated with adverse local effects on the skin, such as irritation or skin atrophy [7]. On the other hand, conventional systemic treatments (such as acitretin, methotrexate, and cyclosporine) and the most recent biological treatments have shown high clinical efficacy. However, they carry risks of liver toxicity, nephrotoxicity, immunosuppression, or serious infections, which restrict their prolonged use [8].

In recent years, Janus kinase (JAK) pathway inhibitors have been considered as a therapeutic alternative for various inflammatory diseases [9,10]. Baricitinib (BCT) is a selective inhibitor of Janus kinases JAK1 and JAK2, whose action blocks the signaling of various cytokines involved in the inflammatory response, such as interleukins (IL-6, IL-12, IL-23) and type I and II interferons. In this way, it prevents the phosphorylation and activation of STATs (Signal Transducers and Activators of Transcription); therefore, BCT interrupts the JAK-STAT cascade responsible for keratinocyte proliferation and immune cell activation [11]. Baricitinib has demonstrated its clinical efficacy in moderate-to-severe psoriasis in a randomized phase 2b trial: patients receiving 8 mg or 10 mg once daily for 12 weeks achieved significantly higher PASI-75 responses compared with placebo (43% and 54% vs. 17%) [12]. However, oral administration of BCT has been associated with significant systemic adverse effects, including an increased risk of opportunistic infections, deep vein thrombosis, and cardiovascular events [13,14]. These limitations have prompted the search for topical formulations of baricitinib that can target the drug at the site of application, reducing systemic exposure and improving the safety profile [12,15,16]. For instance, a topical JAK1/JAK2 inhibitor cream (ruxolitinib phosphate, INCB018424) showed proof-of-concept efficacy in a vehicle-controlled clinical trial for plaque psoriasis, significantly improving lesion thickness, erythema, and scaling compared with a placebo [17]. Although there are very few studies on the topical use of BCT in psoriasis, Bhaskarmurthy DH et al. demonstrated its in vivo efficacy in a psoriasis-like skin inflammation model, as evidenced by the reduction in inflammatory markers [18]. Furthermore, in previous work by our research group, topical BCT formulations using lipid systems were developed. These oily solutions improved drug solubility, promoted cutaneous retention, and demonstrated efficacy in mouse models of psoriasis [19].

The therapeutic efficacy of topical treatments in psoriasis is influenced not only by the active ingredient, but also by the physicochemical properties of the vehicle, which determine skin hydration, barrier modulation and drug penetration [20,21]. Sustained stratum corneum hydration is primarily driven by the reduction in transepidermal water loss through occlusive mechanisms rather than by humectation alone. Thus, stratum corneum hydration (SCH) and transepidermal water loss (TEWL) are complementary biophysical parameters widely used to assess the impact of topical formulations on skin hydration dynamics and epidermal barrier function.

Petrolatum is a classic and widely used excipient in dermatological formulations, thanks to its emollient, occlusive, and moisturizing properties [22]. Liquid petrolatum (LLV) is characterized by its ease of application and good spreadability, while solid petrolatum (LSV) offers greater occlusivity and persistence on the skin [23]. These differences in physical behavior can significantly impact the biopharmaceutical performance of an incorporated drug, particularly in terms of local release and retention. However, to date, no studies have systematically compared the effects of liquid and solid petrolatum in lipid-based BCT formulations.

In this context, this study aims at evaluating the impact of petrolatum type (liquid vs. solid) and its concentration on lipid formulations of BCT solubilized in MCT (Labrafac^®^ Lipophile WL 1349 (L) on skin hydration, epidermal barrier function and the topical delivery and antipsoriatic activity of baricitinib. The incorporation of different petrolatum, such as liquid and semi-solid petrolatum, is expected to generate formulations with graded occlusive properties, enabling a systematic evaluation of their impact on TEWL reduction and stratum corneum hydration.

## 2. Materials and Methods

### 2.1. Materials

**Figure 1:**
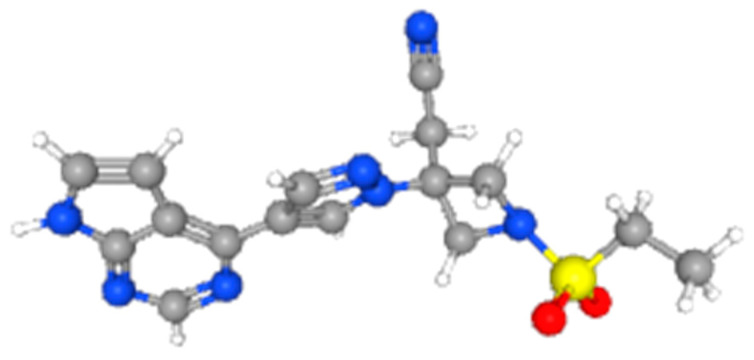
Chemical structure of BCT [24].

Baricitinib [BCT (molecular weight: 371.42 g/mol; molecular formula: C_16_H_17_N_7_O_2_S)] was purchased from Henrikang Biotech Co., Ltd. (Xi’an, China) (Figure 1). Liquid and solid petroleum jelly were purchased from Laboratorios Guinama (Valencia, Spain). Medium-chain triglycerides -MCT Labrafac^®^ Lipophile WL 1349- and Transcutol^®^ were kindly donated by Gattefossé (Saint-Priest, France). PBS Tablets were purchased at Sigma-Aldrich (Madrid, Spain). Imiquimod (IMQ) 50 mg/g cream was obtained from Cantabria Labs (Madrid, Spain). Reagents for histological procedures were purchased from Sigma and Thermo Fisher Scientific (Barcelona, Spain). A Millipore Milli-Q^®^ purification system (Millipore Corporation; Burlington, MA, USA) was used to obtain ultrapure water for all experiments. Finally, the reagents used in this study were of analytical grade.

### 2.2. Experimental Design for the Construction of BCT 0.4 mg/g Formulations

A 2^2^-factorial design was used with two factors: (A) type of petroleum jelly, at two levels [liquid (+) and solid (−)], and (B) concentration of petroleum jelly, at two levels [60% (+) and 30% (−)]. This resulted in four experimental formulations (Table 1; Table 2). A formulation composed of Labrafac™ Lipophile WL 1349 and BCT was prepared and used as a reference control (L).

In all formulations, BCT was solubilized in Labrafac^TM^ Lipophile WL 1349 by constant magnetic stirring at room temperature (25 ± 2 °C) at 300 rpm until a homogeneous solution was achieved. Subsequently, liquid petroleum jelly was added in the established proportion (60% or 30% *w*/*w*), and the mixture was stirred until a homogeneous system was obtained. Solid petroleum jelly was melted at 40 °C and then added to the BCT solution. The control formulation (F5) was obtained by dissolving only BCT in MCT, without the addition of petroleum jelly.

The prepared formulations were allowed to stand for 24 h at room temperature to reach equilibrium. The most suitable formulation was determined based on its physical and chemical stability, which was analyzed 30 and 60 days after preparation under storage conditions of 30 ± 2 °C with 65 ± 5% relative humidity, and 40 ± 2 °C with 75 ± 5% relative humidity.

### 2.3. Characterizations of Formulations

#### 2.3.1. pH

The pH of the formulations was determined using a digital potentiometer (Crison micropH 2000, Crison Instruments SA, Alella, Spain). For the measurement, 1 mL of formulation was dispersed in 20 mL of distilled water, followed by treatment in an ultrasonic bath for 5 min to promote homogeneity [19]. Subsequently, the electrode was then inserted into the sample 24 h after the preparation of the formulations, at a room temperature of 25 ± 2 °C. The results were expressed as mean ± standard deviation (*n* = 3).

#### 2.3.2. Drug Content

The BCT content in the formulations was analyzed according to the methodology described by Mohammadi-Meyabadi et al. [25]. Expressed briefly, to assess the assay of the formulations, samples were diluted in Transcutol^®^ P (diethylene glycol monoethyl ether) as necessary so as to ensure they were within the validated calibration range of the analytical method. BCT concentration was determined by high-performance liquid chromatography (HPLC) using a C18 column, a mobile phase of methanol and water, a flow rate of 1.0 mL/min, and UV detection at 210 nm.

#### 2.3.3. Rheological Properties

Rheological measurements were performed using a Haake Rheostress^®^ 1 rheometer (Thermo Fisher Scientific, Karlsruhe, Germany) equipped with a thermostatic circulator Thermo Haake Phoenix II + Haake C25P (Thermo Electron Corporation, Karlsruhe, Germany). Data acquisition and analysis were performed with Haake Rheowin^®^ Data Manager v. 4.93 software (Thermo Electron Corporation, Karlsruhe, Germany).

Steady-state measurements were carried out with a cone-and-plate geometry (C60/2°Ti: 60 mm diameter, 2° angle). The shear stress (τ) was measured as a function of the shear rate (γ). Viscosity curves (η = f(γ)) and flow curves (τ = f(γ)) were recorded at 25 ± 0.1 °C. The shear rate ramp program included a 3 min ramp-up period from 0 to 100 s^−1^, 1 min constant shear rate period at 100 s^−1^, and 3 min ramp-down from 100 to 0 s^−1^. Data from the flow curves were fitted using mathematical models to identify the model that provided the best overall match of the experimentally observed rheological data: Newton, Bingham, Ostwald de Waele, Herschel–Bulkley, Casson, and Cross with Haake Rheowin^®^ Data Manager v. 4.93 software. The adequacy of the rheological profiles in relation to mathematical models was assessed based on the correlation coefficient value (r) and the chi-square value. Steady-state viscosity (η, mPa·s) was determined from the constant shear section at 100 s^−1^.

#### 2.3.4. Spreadability

The spreadability of the formulations was determined using a laboratory-designed extensometer, which consisted of a fixed base with a movable center connected to a lever. A volume of 400 μL of the BCT formulation was placed in the center of the extensometer, and then a transparent plate with graduations was placed over the sample. When the lever was activated, the movable center rose, compressing the formulation and causing it to expand radially. Weights of 26, 31, 36, 46, 76, and 126 g were applied sequentially, with each load maintained for 1 min. After each application, the extension diameter of the formulation was recorded in centimeters. The determinations were performed in triplicate (*n* = 3), and the results were analyzed and adjusted using mathematical models in GraphPad Prism^®^ v8.0.2 software (GraphPad Software Inc., San Diego, CA, USA).

### 2.4. In Vitro Release Study

Vertical Franz diffusion cells (5 mL) (Franz Diffusion Cells 400; Crown Glass, Somerville, NJ, USA) were used to study the release profile of the BCT formulations. A SpectraPor^®^ regenerated cellulose dialysis membrane (molecular weight cut-off of 14,000 Da; Sigma-Aldrich, Madrid, Spain) was placed between the donor and receptor compartments. Before testing, the membranes were hydrated in a 50:50 (*v*/*v*) mixture of methanol and water for 24 h and subsequently washed with Milli-Q water. The diffusion area size was 0.64 cm^2^. A 50:50 (*v*/*v*) mixture of Transcutol^®^ P/PBS was used to fill the receptor compartment, with a final pH of 7.4. Continuous stirring was applied at 600 rpm.

30 µL of each formulation was deposited in the donor compartment, and the system was maintained at 32 ± 0.5 °C to simulate the physiological conditions of the skin surface. Aliquots of 200 µL of the receptor fluid were extracted at preset time intervals (3, 6, 9, 24, 27, 30, 40, 45, and 50 h), immediately replenished with an equal volume of fresh medium to maintain *sink* conditions. The quantification of the released BCT was performed by HPLC [25]. The results were expressed as the mean ± SD (*n* = 6). For the kinetic analysis, the experimental data were fitted to different mathematical release models, selecting the best-fit model based on the correlation coefficient (r^2^) [26]:

(1) $$\begin{array}{cc} \mathrm{Zero}\;\mathrm{order}\;\;\;\;\;\;\;\;\;\; & Q_{t}=K_{0\cdot t}+Q_{\infty } \end{array}$$

(2) $$\begin{array}{cc} \mathrm{First}\;\mathrm{order}\;\;\;\;\;\;\;\;\;\; & Q_{t}=Q_{\infty }\left(1-e^{-K_{f}\cdot t}\right) \end{array}$$

(3) $$\begin{array}{cc} \mathrm{Second}\;\mathrm{order}\;\;\;\;\;\;\;\;\;\; & \frac{dQ_{t}}{dt}=k_{2}(Q_{\infty }-Q_{t}{)}^{2} \end{array}$$

(4) $$\begin{array}{cc} \mathrm{Higuchi}’s\;\;\;\;\;\;\;\;\;\; & Q_{t}=K_{H}t^{\frac{1}{2}} \end{array}$$

(5) $$\begin{array}{cc} \mathrm{Korsmeyer}\text{–}\mathrm{Peppas}\;\;\;\;\;\;\;\;\;\; & Q_{t}=K_{k}t^{n} \end{array}$$

### 2.5. Ex Vivo Skin Permeation Study

Skin permeation studies were conducted using human skin obtained from abdominoplasty procedures performed on a healthy Caucasian woman (aged 45, normal skin phototype III) who provided written informed consent. The experimental protocol was approved by the Ethics Committee of the Hospital de Barcelona-SCIAS (No. 002, 17 January 2020). The integrity of the samples was verified by determining transepidermal water loss (TEWL) using a Tewameter^®^ TM 300 (Courage & Khazaka Electronics GmbH, Cologne, Germany), accepting only those with values below 10 g/m^2^/h [27]. To obtain 0.4 mm thick sections, human skin samples from abdominoplasty were used and an Aesculap GA 630 dermatome (Aesculap, Tut-193, Tlingen, Germany). The skin samples were then placed between the donor and recipient compartments of vertical Franz diffusion cells (capacity: 5 mL; diffusion area: 0.64 cm^2^). The recipient compartment was filled with a Transcutol^®^ P/PBS solution in a 50:50 (*v*/*v*) ratio and a pH of 7.4. A temperature of 32 ± 2 °C and constant agitation at 600 rpm were applied. In the donor compartment, 30 μL of the 0.04% BCT formulation was applied. The petrolatum-free (F5) formulation was used as a control.

Over 24 h, 200 μL aliquots were collected from the receptor compartment at preset time intervals, with each sample replaced by an equal volume of fresh medium. The concentration of BCT was determined by HPLC [25]. The results were expressed as the mean ± SD (*n* = 6).

At the end of the experiments, the amount of BCT retained in the skin was expressed as micrograms of BCT per gram of skin, normalized by the surface area of the diffusion cell (µg/g skin/cm^2^), and determined by ultrasound-assisted extraction. To do this, the skin samples were removed from the Franz cells, carefully cleaned with gauze pads soaked in a 0.05% sodium dodecyl sulfate solution, and then with distilled water. The exposed area was cut out, weighed, and immersed in 1 mL of Transcutol^®^ P for 30 min in an ultrasonic bath. The extracts obtained were filtered and analyzed via HPLC to determine the amount of drug retained in the skin tissue [25].

### 2.6. Irritation Potential of Formulations: Hen’s Egg Test-Chorioallantoic Membrane

Given that psoriasis may also affect facial regions, including the perioral and perinasal areas, eyebrows, and eyelids (palpebral psoriasis), the HET-CAM test was included in this study to evaluate the potential irritant capacity of the formulations, considering the possible topical application of the formulations on facial areas [28,29] affected by psoriasis, in particular, the regions anatomically close to the eye, eyebrows and eyelids [30,31]. Therefore, assessing the potential for ocular irritation provides relevant safety information.

The Hen’s Egg Test-Chorioallantoic Membrane, also known as HET-CAM, is a method used to evaluate the irritant capacity of chemical substances [32]. The test was performed using chicken eggs incubated for ten days, from which the shell was carefully removed from the part where there is the air cell to reveal the chorioallantoic membrane [33].

300 µL of each formulation was applied to the membrane, and the vascular and albumin responses were recorded during the first 300 s (5 min) after application. The times of onset of hyperemia, hemorrhage, vascular lysis, and clot formation or opacity were noted. Xylene (positive control) and 0.9% sodium chloride solution (negative control) were used as experimental controls (*n* = 3).

The effects were classified according to the time of onset of the reactions observed (Table 3). Finally, the formulations were classified based on the average of the evaluations obtained from the three eggs studied, establishing the following categories: 0.00–4.99 = non-irritating or slightly irritating (NI/SI); 5.00–8.99 = moderately irritating (MI); and 9.00–21.00 = severely irritating (SVI) [34].

### 2.7. Exploratory In Vivo Efficacy Studies: Imiquimod-Induced Psoriasis Model

#### 2.7.1. Animals and Study Protocol

The experimental protocol was approved by the Animal Experimentation Ethics Committee of Bioterio-UAEM at the Universidad Autónoma del Estado de Morelos (number 2023003 with approval on 12 May 2023). It was conducted in accordance with current animal welfare regulations, the Mexican Official Standard NOM-062-ZOO-1999 [36] for animal care, and the ARRIVE guidelines. The study was designed to evaluate the efficacy of BCT formulations in treating skin inflammation induced in a murine model of psoriasis. In this exploratory phase, the objective was to determine whether the formulations exhibited sufficient anti-psoriatic activity to justify further development. The decision criteria included a clear observable reduction in erythema, scaling, and skin thickening. Four- to five-month-old BALB/c mice were used, housed in three separate cages according to the experimental group (*n* = 5 per group), in a room with controlled temperature and humidity conditions, with free access to food and water (ad libitum).

The animals were randomly assigned to three groups: negative control, positive control, and a treatment group. The negative control group consisted of healthy animals that did not receive any topical application (neither imiquimod [IMQ] nor formulation). In both the positive control and treatment groups, psoriasis was induced by applying IMQ (5 mg/mL) to the back and right ear, administered once daily for six consecutive days. Subsequently, the animals in the positive control group received 50 µL of PBS once daily for an additional six days, while those in the treatment group received 50 µL of the BCT formulation under the same administration schedule.

**Figure 2:**
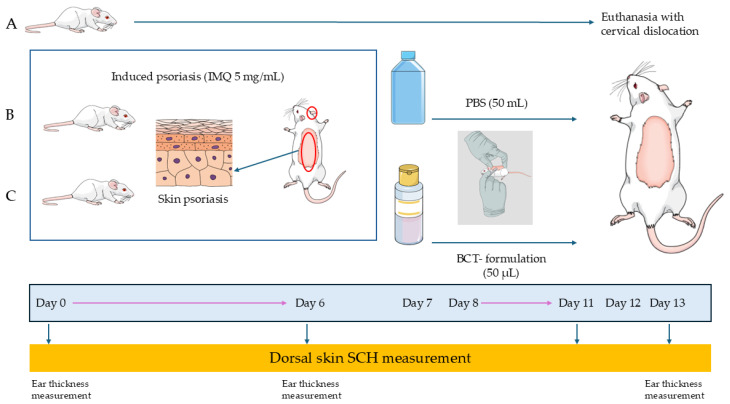
Experimental design in mice with induced psoriasis and topical treatment with baricitinib. (**A**) Negative control; (**B**) Positive control; (**C**) BCT-Formulations.

Finally, the animals were carefully sacrificed by cervical dislocation, and skin samples were taken for histological analysis (Figure 2).

#### 2.7.2. Skin Thickness Assessment

The ear thickness of the mice in each experimental group was determined throughout the study using a portable ear thickness gauge model 7309 (Mitutoyo Corp., Kawasaki, Japan). Measurements were taken at different times: on day 0 (start), day 6 (peak swelling), and day 13 (end of the trial). Likewise, after the animals were sacrificed, the dorsal skin thickness was measured using the same method.

#### 2.7.3. Biomechanical Skin Properties Evaluation

Throughout the experimental protocol, biomechanical parameters of the mouse skin were examined, assessing transepidermal water loss (TEWL) and the stratum corneum hydration level (SCH). The CM-825 Corneometer (Courage & Khazaka Electronics GmbH, Cologne, Germany) was used to determine SCH, and the DermaLab1 Tewameter module (Cortex Technology, Aalborg, Denmark) was used to measure TEWL. These parameters were recorded at predetermined time points: on day 0 (onset), on day 6 (maximum inflammation), and on days 8, 11, and 13. This was performed to observe fluctuations over time in the skin barrier function and the hydration status.

#### 2.7.4. Histological Analysis

Skin samples collected from the dorsal region and ear of mice were fixed with 4% formaldehyde for 24 h at 2 °C. They were then washed three times with PBS, changing the medium every hour. The samples were gradually dehydrated in ethanol solutions of increasing concentration and then clarified with xylene. Finally, the skin samples were embedded in paraffin blocks and cut into 5 μm-thick sections, which were then stained with hematoxylin and eosin for subsequent histological analysis using an optical microscope (Olympus BX41, Olympus Co., Tokyo, Japan) equipped with a digital camera (Olympus XC50, Olympus Co., Tokyo, Japan).

Epidermal thickness was quantified by histomorphometric analysis using digital micrographs of hematoxylin and eosin (H&E)–stained skin sections obtained from dorsal and ear samples. Calibration was performed using the embedded scale bar (100 µm for dorsal skin and 50 µm for ear skin) before measurement. The epidermal thickness was defined as the perpendicular distance between the dermo–epidermal junction and the upper limit of the stratum corneum, excluding the stratum corneum when partially detached. Measurements were carried out manually at ten points along each section using ImageJ software version 1.52v (NIH, Bethesda, MD, USA) after scale calibration. The mean epidermal thickness (mean ± SD) was calculated for each image and used for statistical analysis.

### 2.8. Tolerance Study

#### 2.8.1. Tolerance Study in the Dorsal Skin of the Mouse

The in vivo tolerance of the LSV and LLV formulations was evaluated in BALB/c mice, following the guidelines established by the Ethics Committee of the Bioterio-UAEM of the Universidad Autónoma del Estado de Morelos (number 2023004, approved on 12 May 2023). The study design and reporting adhered to the ARRIVE guidelines. The study included four experimental groups: (i) a negative control (untreated animal), (ii) a positive control (xylene-treated), and the topically treated groups (iii) with the LSV, and (iv) LLV formulations. In all cases, 50 μL of the corresponding formulation or xylene was applied to the previously shaved dorsal skin. After 180 min of exposure, the animals were sacrificed by cervical dislocation, and skin samples were taken for histological analysis.

#### 2.8.2. Occlusive Effect of the Formulation’s Excipients on the Skin of Healthy Volunteers

For this test, defined areas on the anterior surface of the forearm were selected on ten healthy volunteers, who participated after giving their informed consent, in accordance with the Declaration of Helsinki and approved by the Ethics Committee of the University of Barcelona (number IRB00003099; approval issued on 20 March 2018). Inclusion criteria included the presence of healthy skin on the volar forearm, age between 18 and 60 years old, both genders, male and female, and absence of any dermatological or systemic condition that could interfere with the study results. Exclusion criteria were pregnancy or lactation, ongoing dermatological treatment, and the use of systemic anti-inflammatory or immunosuppressive medication.

As a prerequisite, participants were required to refrain from applying any cosmetic or topical product for at least 12 h prior to the test so as to avoid interference with the skin barrier function.

The formulations tested did not contain BCT and were applied in an amount of 0.5 mL/cm^2^ to the epidermal surface defined for each volunteer. An untreated area was maintained as a negative control.

TEWL and SCH were determined, enabling the noninvasive measurement of water vapor flux through the skin and skin hydration, expressed in g/m^2^/h and AU, respectively. Measurements were recorded at the following time points: 0 (baseline), 10, 20, 30, 60, 120, and 180 min after the application of the formulations. A water–Transcutol solution was included as a non-occlusive reference vehicle, representing a volatile and penetration-enhancing system lacking film-forming properties.

## 3. Results

To investigate the impact of excipient composition on the topical delivery of baricitinib, a series of lipid-based formulations were developed, as detailed in Section 2.2. These formulations incorporated MCT as a vehicle, combined with either solid or liquid petrolatum at varying concentrations. The selected excipients were chosen for their distinct physicochemical properties, particularly their occlusive and emollient effects, which are known to influence drug release, skin permeation, and retention. The concentration of 0.04% (*w*/*w*) baricitinib used in this study was selected based on its solubility in the chosen excipients [25]. Although this specific concentration has not been previously reported for topical application, similar concentrations, such as 0.05%, have been described in the literature by Nene et al. [37]. The following results present the comparative evaluation of the formulations in terms of their stability, rheological behavior, spreadability, in vitro release, ex vivo permeation, and skin deposition profiles.

### 3.1. Experimental Design for the Construction of BCT 0.4 mg/g Formulations

The formulations developed were subjected to accelerated storage conditions at 30 ± 2 °C and 65 ± 5% RH (Table 4) and 40 ± 2 °C and 75 ± 5% RH (Table 5) for a period of 60 days. During this interval, drug precipitation was observed from day 35 onwards in formulations LSV_1_ and LLV_1_ stored at 30 °C; therefore, these samples were excluded from further evaluation. In contrast, LSV_2_ and LLV_2_ formulations maintained a homogeneous and stable appearance throughout the 60 days under the established storage conditions.

In the case of LSV_1_ and LLV_1_, the pH and drug content (% assay) parameters were monitored only up to day 30, as precipitation prevented further analysis. In contrast, formulations LSV_2_ and LLV_2_ showed more robust behavior at 30 ± 2 °C/65 ± 5% RH, both maintaining stable pH values (5.6–5.8) and drug content above 96% after 60 days of storage. Similarly, at 40 ± 2 °C and 75 ± 5% RH, the same formulations retained their physicochemical stability, with minimal variations in pH (%RE: −8.47%) and drug content above 95% at the end of the study.

Formulations with 60% petrolatum (LLV1 and LSV1) exhibited partial precipitation and phase separation after 35 days, while those containing 30% petrolatum (LLV2 and LSV2) remained homogeneous and stable in color and texture throughout the 60-day period.

Physical inspection was conducted during the storage to assess appearance, color, and phase separation. Formulations containing 60% petrolatum (LLV1 and LSV1) exhibited turbidity and partial precipitation from day 35 onward, indicating reduced physical stability under accelerated conditions. In contrast, formulations with 30% petrolatum (LLV2 and LSV2) maintained homogeneity, consistent color, and no evidence of phase separation throughout the 60-day storage period.

The formulations selected for characterization were those composed of 30% petrolatum (solid or liquid), 69.96% MCT, and 0.04% Baricitinib. From this point on, these formulations were referred to as LSV (formulation with solid petroleum jelly) and LLV (formulation with liquid petroleum jelly), respectively.

First-order degradation kinetics were observed for the stable formulations LSV2 and LLV2 (Table 6). The determination coefficients (r^2^ > 0.94) indicated a good fit to the first-order model. As expected, degradation accelerated with temperature: at 30 °C/65% RH, t_90_ values were 151.98 days (LSV2) and 124.03 days (LLV2); at 40 °C/75% RH, they decreased to 93.46 days and 75.17 days, respectively. The rate constants (k) roughly doubled with each 10 °C increase, consistent with Arrhenius behavior. Formulations containing 60% petrolatum (LSV1, LLV1) showed phase separation after 35 days and were not included in the kinetic comparison. Statistically significant differences (*p* < 0.001) were observed in the degradation rate constant (k) between storage temperatures for both formulations. For LLV2, the k value increased from 0.08062 day^−1^ at 30 °C to 0.13303 day^−1^ at 40 °C, and for LSV2, from 0.06580 day^−1^ at 30 °C to 0.10700 day^−1^ at 40 °C, indicating faster degradation at higher temperature. In addition, significant differences (*p* < 0.001) were also detected between formulations at the same temperature, with LSV2 showing lower degradation rate constants than LLV2 at both 30 °C and 40 °C.

### 3.2. Characterizations of Formulations

#### 3.2.1. Rheological Properties

**Figure 3:**
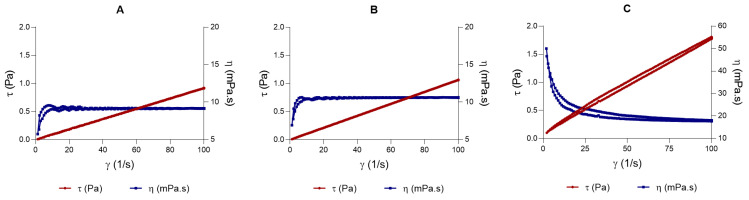
Rheological behavior of baricitinib formulations analyzed under rotational conditions at 25 ± 0.1 °C. (**A**) BCT in MCT; (**B**) LLV formulation and (**C**) LSV formulation. The red line represents the flow curve, and the blue line represents the viscosity curve.

Figure 3 presents the steady-state rheological measurements according to shear rate. Formulations L and LLV exhibited Newtonian rheological behavior, with a constant viscosity and a linear flow curve. No thixotropy was observed. LSV exhibited pseudoplastic flow, showing a consistent decrease in viscosity with increasing shear rate from 0 to 100 s^−1^. The viscosity values at 100 s^−1^ were 9.16 ± 0.01, 10.62 ± 0.01, and 17.88 ± 0.09 mPa·s for L, LLV, and LSV, respectively. The measurements were reproducible with a coefficient of variation below 5%.

Regarding viscosity measurements, at 100 s^−1^, LSV showed a higher viscosity value, while formulations L and LLV had the lowest viscosity values compared to LSV. The LSV data were fitted to the Cross equation, indicating pseudoplastic behavior, unlike the Newtonian model observed for LLV and L (Table 7). Table 8 presents the parameter values predicted by the best-fitting rheological model.

#### 3.2.2. Spreadability

As for the maximum spreadability values, it was observed that the LLV formulation had the highest spreadability (771.52 ± 78.23 cm^2^), which is attributable to its low viscosity, facilitating greater displacement of the product under pressure. The L formulation, composed solely of Labrafac™ Lipophile WL 1349, also showed high spreadability (685.15 ± 36.45 cm^2^), confirming that this lipid vehicle allows adequate dispersion of the drug. In contrast, the LSV formulation exhibited significantly lower spreadability (138.36 ± 24.02 cm^2^), which is attributed to the greater consistency and structural rigidity provided by this excipient, thereby limiting the product’s spread on the surface.

**Figure 4:**
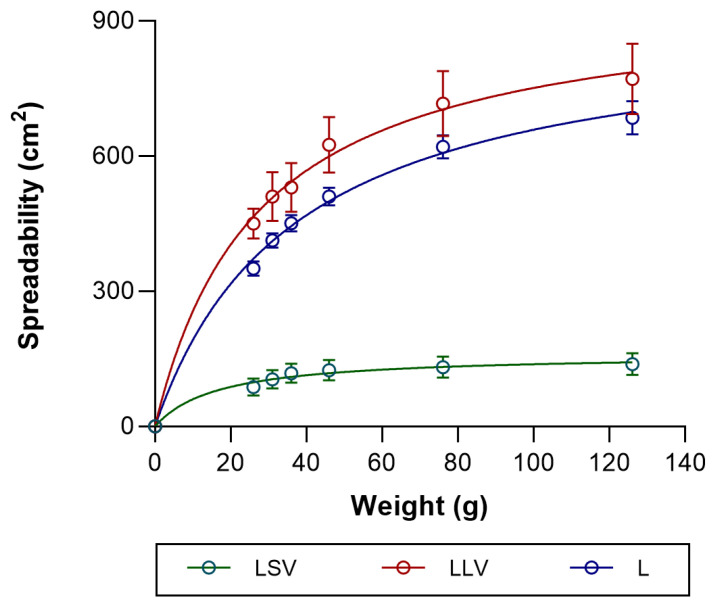
Spreadability profile of BCT formulations. The spreadability followed a hyperbolic model. Values represent mean ± SD (*n* = 3).

The formulations studied showed a spreadability profile that fit the hyperbolic mathematical model, yielding coefficients of determination (r^2^) of 0.99, 0.96, and 0.86 for the L (control), LLV, and LSV formulations, respectively (Figure 4). These values suggest a good fit of the model, particularly in the case of the control formulation and the one containing liquid petroleum jelly. The model predicted the maximum spreadability value (Smax) of 902.4 ± 28.2 cm^2^ for L, 957.7 ± 54.3 cm^2^ for LLV and 161.0 ± 16.0 cm^2^ for LSV, with spreading constants (Ks) of 37.01 ± 2.86 g, 27.45 ± 4.54 g and 16.68 ± 6.28 g for L, LLV and LSV, respectively, corresponding to the half-maximum spreading weight, that is, the weight required to achieve half of the maximum extensibility. A higher value of Smax indicates that the formulation can spread over a larger surface area under the applied load, which typically reflects lower resistance to deformation and potentially better skin coverage.

The statistical analysis of the spreading parameters confirmed significant differences among the three formulations. For the maximum spreadability (Smax), one-way ANOVA showed highly significant differences (*p* < 0.0001), with both L and LLV displaying markedly higher values than LSV (*p* < 0.001), while no significant difference was observed between L and LLV. Similarly, the spreading constant (Ks) differed significantly among formulations (*p* = 0.0059), with L showing a higher Ks than LSV (*p* < 0.01). These findings indicate that the formulations containing liquid oily excipients exhibit greater overall extensibility. In contrast, the LSV formulation, which incorporates solid petrolatum, shows a markedly lower Smax and limited deformation capacity, consistent with its higher viscosity and more rigid semisolid structure.

### 3.3. In Vitro Release Study

**Figure 5:**
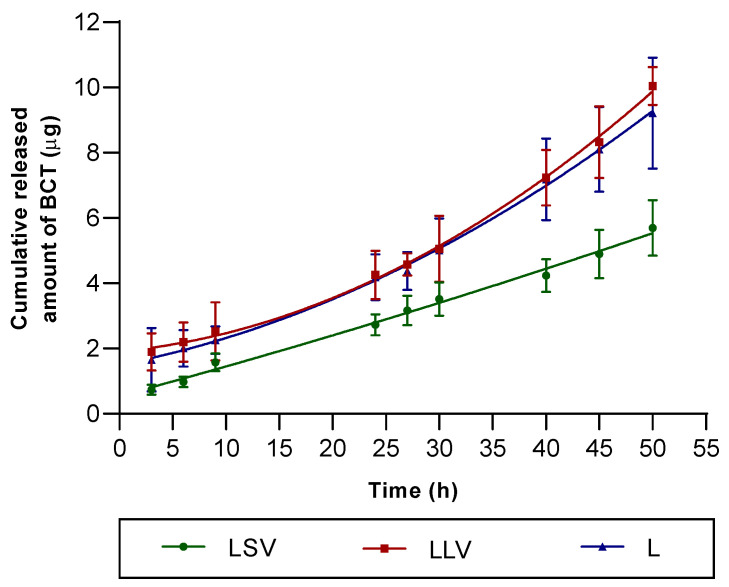
In vitro drug release profile of BCT formulations. Values represent mean ± SD (*n* = 6).

The release profile of BCT from the different formulations was represented as the cumulative amount released as a function of time (Figure 5). The results showed significant differences in the release kinetics among the evaluated formulations. After 50 h of testing, the cumulative release was 47.43 ± 10.02% for the LSV formulation, 83.73 ± 6.84% for LLV, and 76.75 ± 20.03% for L, suggesting that the incorporation of solid petroleum jelly decreases the release of the active. In contrast, the addition of liquid petroleum jelly may promote the release of the active ingredient comparable to the MCT.

The release profiles exhibited a second-order polynomial model, indicating that drug release did not follow strict linear kinetics but rather presented distinct phases. Specifically, the cumulative release increased progressively over time. The fit was confirmed by the coefficients of determination (r^2^), which reached values of 0.94 for LSV, 0.95 for LLV, and 0.91 for L, showing a high degree of correlation between the experimental data and the mathematical model (Table 9).

### 3.4. Ex Vivo Permeation Study

**Figure 6:**
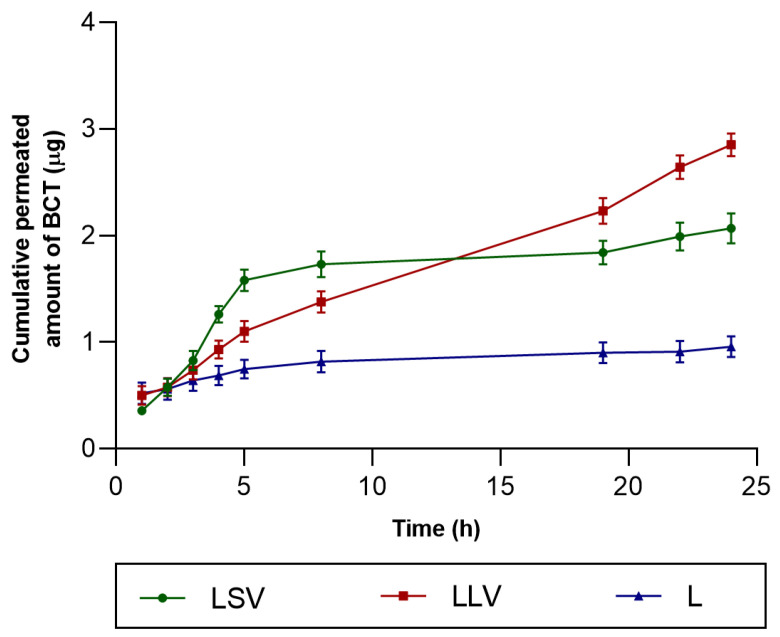
Ex vivo drug permeation profile of BCT formulations. Values represent mean ± SD (*n* = 6).

Figure 6 shows the results of ex vivo permeation studies of BCT through human skin using the different formulations developed.

The integrity of each skin disc used for the IVPT study was verified before mounting in the Franz diffusion cells by measuring transepidermal water loss (TEWL). In addition, TEWL was measured again at the end of the experiment to confirm that exposure to the receptor medium did not compromise the integrity of the skin barrier. Table 10 summarizes the transepidermal water loss (TEWL) values measured at the beginning and at the end of the permeation studies for each formulation (L, LLV, and LSV). Values remained within the accepted range (<10 g/h/m^2^), confirming that the integrity of the skin barrier was maintained throughout the assays.

The amount of drug retained in the skin after 24 h showed different behaviour: LLV (94.05 ± 17.87 µg/g of skin/cm^2^) retained the highest amount, followed by L (84.18 ± 15.15 µg/g of skin/cm^2^) and finally LSV (49.72 ± 8.95 µg/g of skin/cm^2^) (Table 11). Although LSV facilitated a more efficient permeation at early times into the receptor compartment (Qp_5_), LLV promoted a higher permeation after 24 h, and a greater drug deposition within the skin matrix at the end of the study. Drug retention in the skin is an advantage for local topical application with a sustained effect on the skin.

### 3.5. Irritation Potential of Formulations: Hen’s Egg Test-Chorioallantoic Membrane

**Figure 7:**
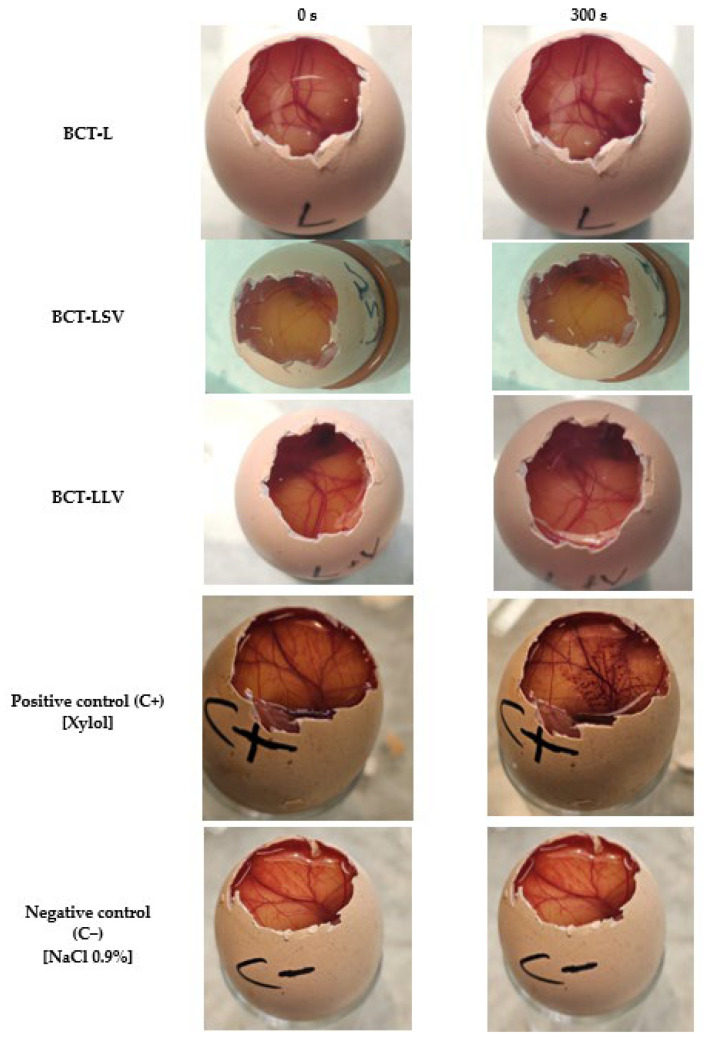
Photographs of HET-CAM test results of the BCT formulations to assess the potential ophthalmic irritancy.

To assess the potential irritant effect of the formulations, the HET-CAM test was performed. This test was selected so as to evaluate the suitability of the formulations for facial application on areas commonly affected by psoriasis, in particular those zones close to the eyes, such as the eyebrows and eyelids. HET-CAM assays did not reveal any macroscopic alterations in the chorioallantoic membrane after exposure to formulations containing BCT (Figure 7). In accordance with the scoring-based classification system (the reader is referred to Section 2.6), all formulations were categorized as non-irritating (NI). Therefore, the preparations evaluated have an adequate safety profile for topical application, as they do not induce immediate irritative reactions in the model used.

### 3.6. Exploratory In Vivo Efficacy Studies: Imiquimod-Induced Psoriasis Model

**Figure 8:**
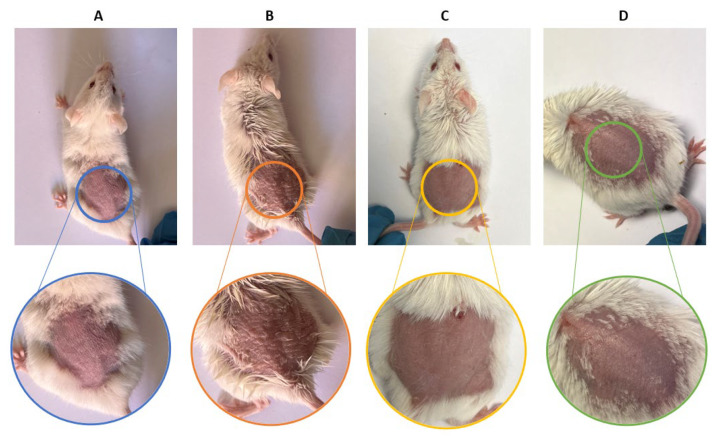
Photographs of the dorsal skin of mice were taken at the end of the experiment to evaluate the efficacy of the formulations on psoriasis induced by imiquimod: (**A**) Negative control group; (**B**) Positive control group; (**C**) LSV-treated group; and (**D**) LLV-treated group.

Topical administration of IMQ to the dorsal skin of mice for six days resulted in the appearance of erythematous lesions, scaling, and edema, clinical features representative of psoriasis compared to the negative control group (Figure 8A,B). However, daily treatment with the formulations evaluated over the same period significantly reduced these signs, demonstrating a progressive improvement in skin appearance compared to the positive control group (Figure 8C,D).

#### 3.6.1. Biomechanical Properties and Skin Thickness Evaluation

**Figure 9:**
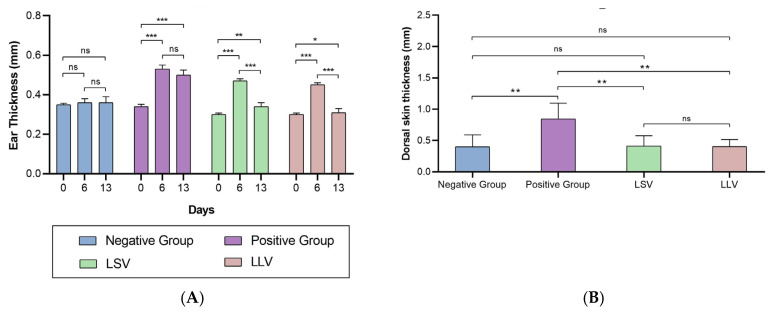
Skin thickness assessment in (**A**) auricular tissue and (**B**) dorsal skin. Psoriasis-like lesions were induced by the topical application of imiquimod in both the positive control group and the LSV and LLV-treated group. Values represent the mean ± SD (*n* = 5). Statistical significance was determined as follows: * *p* < 0.05, ** *p* < 0.01, *** *p* < 0.001; ns = not significant.

Following skin inflammation by topical application of IMQ for six days, a significant increase in auricular thickness was recorded in mice compared to baseline values. However, topical administration of BCT formulations (LSV and LLV) for six days after psoriatic induction produced a statistically significant reduction in this parameter, demonstrating a therapeutic effect in the attenuation of dermal edema (Figure 9A). Analysis of dorsal skin thickness (Figure 9B), performed after euthanasia of the animals, confirmed that the positive control group exhibited marked skin thickening. In contrast, BCT-treated animals showed values comparable to those observed in the negative control group.

**Figure 10:**
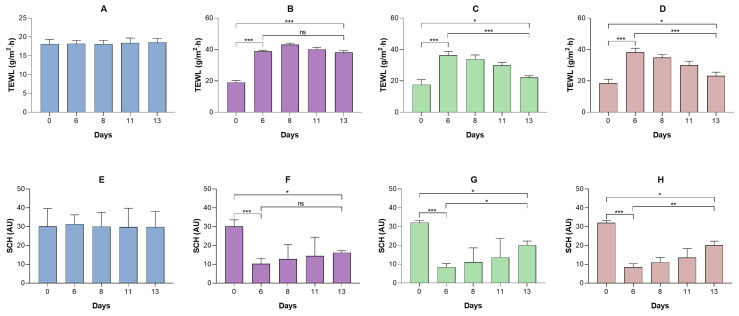
Evaluation of the biomechanical properties of the skin in the different experimental groups of the imiquimod-induced psoriasis model. Transepidermal water loss (TEWL) was determined in the (**A**) negative control group; (**B**) the positive control group; (**C**) the group treated with the LSV formulation; (**D**) and the group treated with the LLV formulation. Additionally, stratum corneum hydration (SCH) was evaluated in the same groups: (**E**) negative control, (**F**) positive control, (**G**) LSV, and (**H**) LLV. Results are expressed as mean ± standard deviation (*n* = 5). Statistical analysis was performed using one-way ANOVA followed by Dunnett’s post hoc test, comparing: (i) the value obtained after psoriasis induction with imiquimod (day 6) versus the basal value; (ii) the value at the end of the treatment versus the basal value; and (iii) the value at the end of the treatment versus the value after psoriasis induction. Statistical differences are indicated as follows: * *p* < 0.05; ** *p* < 0.01; *** *p* < 0.001; and *ns*, not significant.

Topical application of IMQ to the dorsal skin of mice produced a significant increase in transepidermal water loss and a decrease in the water content of the stratum corneum, reflecting a deterioration in the integrity of the skin barrier; these changes correlated with the evident dryness and formation of scaly lesions observed macroscopically from the sixth day of treatment onwards (Figure 10). Furthermore, animals treated topically with the developed formulations showed a significant reduction in TEWL values and an increase in SCH levels, demonstrating a partial restoration of the skin barrier properties.

#### 3.6.2. Histological Analysis

**Figure 11:**
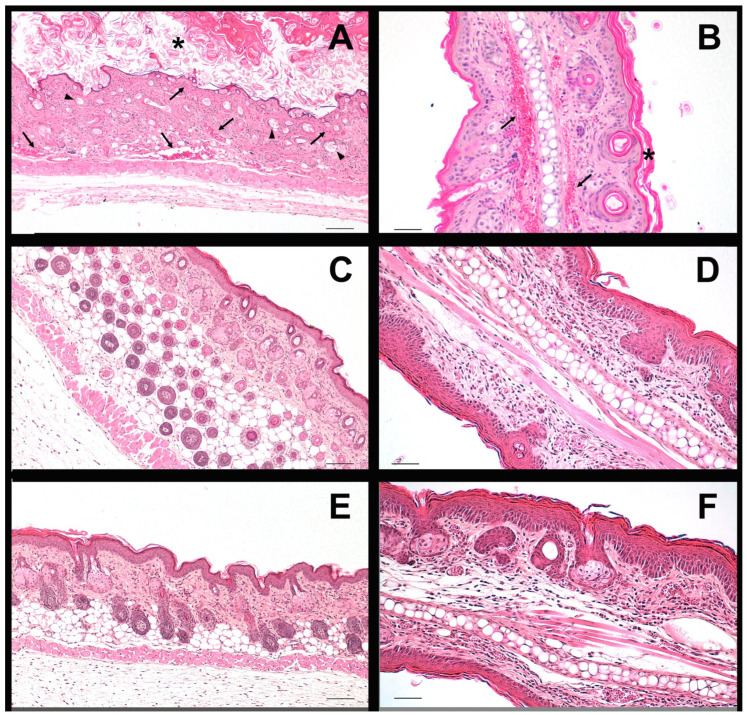
Histological images of the mouse dorsal (**A**,**C**,**E**) and ear skin (**B**,**D**,**F**). (**A**,**B**) positive control group (**C**,**D**) skin of the group treated with the LSV formulation; (**E**,**F**) skin of the group treated with the LLV formulation; (The asterisks stand for increased thickness, arrows indicate vascular dilation characteristics of inflammatory processes, and arrowheads denote poorly structured hair follicles. Scale bars represent 100 µm (**A**,**C**,**E**) and 50 µm (**B**,**D**,**F**).

To assess the potential impact of the topical formulations on skin integrity, histological analyses were performed on mice. The skin positive control (Figure 11A), treated with imiquimod, shows increased thickness of the stratum corneum of the epidermis (*), with loosely attached cells and poorly organized subcorneal layers. The dermis is highly vascularized, containing numerous blood vessels filled with blood (arrows) and hair follicles that appear poorly structured (arrowheads). These features are consistent with an inflamed skin appearance.

In contrast, skin samples treated with either product LSV (Figure 11C) or the product LLV (Figure 11E) display a well-organized epidermis with clearly defined layers. The dermis appears normal, with both blood vessels and hair follicles maintaining their structural integrity and showing no apparent signs of inflammation.

Similar patterns were observed in ear samples treated with imiquimod (Figure 11B), either alone or in combination with formulation LSV (Figure 11D) or LLV (Figure 11F). Ears treated with imiquimod alone exhibited a thicker and partially detached stratum corneum (*), along with a thinner stratum spinosum. The dermis was highly vascularized (arrows) compared to the ears also treated with products LSV and LLV.

Histological quantification of the viable epidermal thickness in dorsal skin revealed a significant reduction in the groups treated with LSV and LLV compared to the imiquimod control (Table 12), indicating partial normalization of epidermal hyperplasia. In contrast, no statistically significant differences were observed in ear skin, where epidermal thickness remained similar across groups. Although ears treated with imiquimod alone exhibited clear signs of inflammation (characterized by dermal vascular dilation and congestion (arrows)), the epidermal layer remained relatively thin and well-preserved, resulting in similar epidermal thickness values across groups.

### 3.7. Tolerance Study

#### 3.7.1. Tolerance Study in the Dorsal Skin of the Mouse

**Figure 12:**
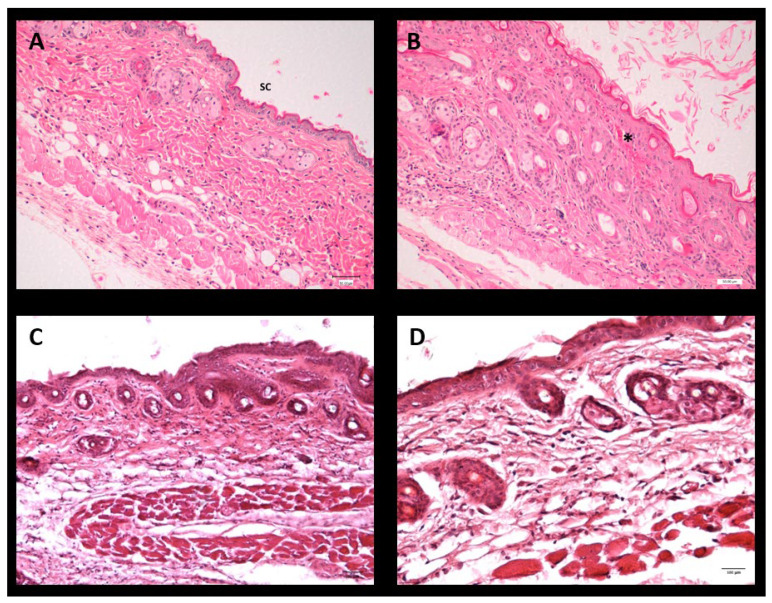
Histological photographs of dorsal skin tissue from mice after 2 h of treatment. (**A**) Negative control group; (**B**) positive control group treated with xylene; (**C**) LLV-treated group; (**D**) LSV-treated group. The stratum corneum (SC) is identified, and the asterisk (*) indicates areas of stratum corneum and epidermis loss—scale bar: 100 µm.

The skin tolerability of the LSV and LLV formulations was evaluated in a mouse model and compared with both the negative control group (untreated animals) and the positive control group (animals exposed to xylene to induce inflammation). Histological analysis of the negative control revealed a normal skin structure, characterized by a relatively thin epidermis and a continuous and intact stratum corneum (SC) (Figure 12A). Furthermore, topical application of xylene in the positive control produced marked epidermal disruption with partial loss of the SC and structural alterations associated with acute inflammation (Figure 12B). It was also shown that skin treated with the study formulations presented histological characteristics comparable to those of the negative control, with no evidence of tissue damage or signs of inflammatory reaction (Figure 12C,D).

#### 3.7.2. Occlusive Effect of the Formulation’s Excipients on the Skin of Healthy Volunteers

**Figure 13:**
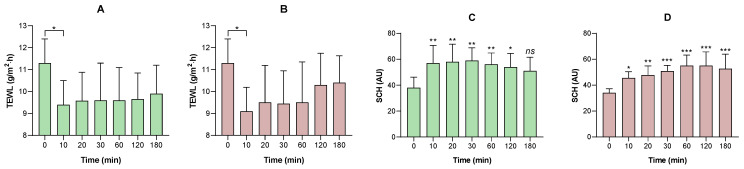
Skin tolerance in humans: (**A**) transepidermal water loss (TEWL) of LSV; (**B**) TEWL of LLV; (**C**) stratum corneum hydration (SCH) of LSV; (**D**) SCH of LLV. Data expressed as mean ± SD (*n* = 10). Statistical analysis was performed using one-way ANOVA followed by Dunnett’s post hoc test, comparing each time point with the corresponding basal value. Significance: * *p* < 0.05; ** *p* < 0.01; *** *p* < 0.001; *ns* = not significant.

Figure 13A,B show a statistically significant reduction in transepidermal water loss after 10 min of topical application of the LSV and LLV blank formulations. However, a progressive increase in this parameter was observed after 15 min, reaching values close to baseline as time progressed. Regarding stratum corneum hydration, Figure 13C,D, the LLV blank formulation produced a significant increase compared to baseline at all intervals analyzed (10, 15, 30, 60, 120, and 180 min), while the LSV blank formulation lost statistical significance at 180 min. In both cases, a general trend toward a return to baseline conditions was evident towards the end of the experiment. During the evaluation, the volunteers did not report itching or pain, confirming the good cutaneous tolerability of both formulations.

The formulations were compared to a non-occlusive vehicle to evaluate the impact of the vehicles on the SCH and TEWL values. Figure S1 shows the evolution of SCH and TEWL following topical application of the formulations, expressed as percentage variation with respect to baseline values. Regarding SCH (Figure S1A), all MCT-based formulations induced a progressive increase in hydration over time. TEWL measurements (Figure S1B) revealed a formulation-dependent reduction in water loss. The strongest decrease in TEWL was observed for LSV, followed by LLV and L.

## 4. Discussion

In this work, we developed lipid-based topical formulations containing baricitinib at a concentration of 0.04% for the treatment of psoriasis. Baricitinib, a selective JAK1/JAK2 inhibitor, has demonstrated clinical efficacy in systemic administration for inflammatory skin diseases; however, topical delivery may represent a safer and more targeted therapeutic approach by minimizing systemic exposure. The rationale for exploring the topical route is supported by prior success with other JAK inhibitors used dermally. For instance, ruxolitinib cream, used to treat atopic dermatitis and vitiligo [16,35], is one example. Studies by Naeimifar A. et al. developed a topical nanoliposomal formulation of this same active ingredient, demonstrating optimal physicochemical properties; however, the study did not demonstrate in vivo efficacy [38]. Nevertheless, clinical trials have been conducted with ruxolitinib cream for the treatment of psoriasis. In this study, patients received a vehicle without an active ingredient and a vehicle with ruxolitinib phosphate at 0.5% and 1.0% once daily, or at 1.5% twice daily, for 28 days. Additional groups included two active comparators: calcipotriol cream 0.005% or betamethasone dipropionate cream 0.05%. The results showed that both the 1% and 1.5% creams improved lesion thickness, erythema, and scaling and reduced their area compared to placebo [17].

Other JAK1 inhibitors have also been used to be delivered in cream form for the treatment of psoriasis, such as Brepocitinib which was used to test its efficacy in cases of mild or moderate plaque psoriasis in a randomized phase IIb clinical study, however, the results were not very encouraging since although the cream was well tolerated, it did not produce statistically significant changes compared to the vehicle without the drug [39].

Finally, a randomized, phase IIb clinical trial of tofacitinib ointment (a JAK1/JAK3 inhibitor and some JAK2 inhibitors) was conducted in patients with plaque psoriasis for 12 weeks. The study demonstrated that the 2% ointment, applied once or twice daily, showed greater efficacy than the vehicle-free ointment at week 8, suggesting that the topical route of a JAK inhibitor may offer a promising alternative for the treatment of psoriasis. However, the lack of statistically significant differences among many regimens at week 12 raised concerns about the magnitude and duration of the clinical effect under the conditions of this study [40]. Nevertheless, the very low systemic exposure and acceptable tolerability profile with the vehicle used support the safety of short-term topical application as shown in this study.

The results obtained demonstrate that the type of petrolatum, whether solid (LSV) or liquid (LLV), exerts a decisive effect on the release, permeation, cutaneous retention, and biological activity of BCT. Following the experimental design, it was indicated that only formulations with 30% petrolatum (LSV2 and LLV2) showed adequate physicochemical profiles over 60 days (Table 4; Table 5), confirming that the selection of the vehicle not only determines the release properties but also the medium-term stability, a critical aspect in lipid formulations [41]. On the other hand, the pH values of the formulations were maintained between 5 and 6, which is ideal for skin application. This is important since the skin barrier and microbiota balance must be maintained to prevent skin irritation and sensitivity [42]. The chemical stability of baricitinib within the topical lipid-based formulations was satisfactory under both intermediate (30 °C/65% RH) and accelerated (40 °C/75% RH) storage conditions. The estimated t_90_ values (ranging from approximately 75 to 152 days) indicate that the formulations maintained at least 90% of their initial drug content for periods exceeding two to five months, depending on temperature. The rate constants (k) approximately doubled with each 10 °C increase, in agreement with the Arrhenius equation, suggesting that the degradation process is thermally activated.

Rheology in topical formulations is not just a physical-chemical parameter; it is crucial for the product’s stability, efficacy, and clinical acceptability [43]. The LSV exhibited pseudoplastic behavior and higher viscosity, whereas LLV displayed a Newtonian profile with reduced viscosity. These differences directly influence spreadability: the LLV formulation disperses more easily over the surface, which is advantageous for topical application on large or sensitive areas. In contrast, LSV, with greater structural rigidity, exhibited lower spreadability, which may limit application uniformity but enhance occlusivity and, therefore, transdermal penetration [44,45].

Spreadability is an essential parameter in topical formulations, as it determines their ease of application and skin coverage. In this study, LLV was favored due to its greater spreadability, which is attributed to its low viscosity, thereby optimizing dispersion and clinical acceptability. On the other hand, LSV exhibited lower spreadability, which is associated with its structural rigidity, although it may have a potential occlusive effect that could benefit skin penetration. Thus, the balance between spreadability and occlusivity is decisive for therapeutic efficacy [46]. A relationship was observed between the rheological and spreadability characteristics of the formulations and the amount of baricitinib released in vitro. The formulation with lower viscosity and greater extensibility (LLV) showed a higher cumulative drug release compared to the more viscous system (LSV), suggesting that softer and more easily spreadable vehicles facilitate drug diffusion through the matrix. This finding is consistent with previous works showing an inverse correlation between viscosity and drug release from semisolid systems [47,48]. The rheological behavior determines the internal structure and mobility of the vehicle, which directly influences diffusion and drug availability. Higher viscosity impedes drug diffusion by creating a denser matrix, reducing the mobility of the drug molecule [49].

The in vitro release test was conducted to evaluate the release profile of baricitinib from the formulation independently of the skin barrier. Therefore, an inert synthetic membrane (dialysis membrane, MWCO 14 kDa) was selected to act merely as a physical support separating the formulation from the receptor medium, without influencing drug diffusion. This approach complies with the recommendations of the EMA Guideline on quality and equivalence of locally applied and locally acting cutaneous products (EMA/CHMP/QWP/708282/2018) [49], which distinguish between in vitro release test —where an inert membrane is required—and in vitro permeation studies, where human or animal skin should be employed to evaluate permeation through the stratum corneum. The in vitro release study showed that liquid petrolatum (LLV) favors a higher and sustained release of BCT compared to solid petrolatum, reaching more than 80% of the drug released at 50 h (Figure 5). This finding is consistent with previous work in which fluid lipid vehicles increase the molecular mobility of the active ingredient and facilitate its diffusion to the skin surface [19]. According to the Korsmeyer–Peppas model, the diffusion exponent (n) is an empirical parameter that characterizes the mechanism of drug release from a semisolid or polymeric matrix. It reflects the relative contribution of Fickian diffusion and relaxation or erosion processes within the vehicle, thus providing insight into whether the release follows purely diffusional, anomalous (non-Fickian), or zero-order kinetics. The observed Korsmeyer–Peppas exponent values (*n* = 0.73–0.78) for our Labrafac- and petrolatum-based ointments are consistent with anomalous (non-Fickian) transport, indicating a combined contribution of diffusion and time-dependent changes at the vehicle interface. This behaviour has been reported specifically for oleaginous ointment bases, where a transient interfacial boundary layer and dynamic microstructural rearrangements govern the release rather than pure Higuchi-type diffusion. Experimental and review studies on ointments and other non-swelling lipidic matrices have emphasised that release kinetics from such bases frequently deviate from simple diffusion models and are better interpreted through models that allow mixed mechanisms (e.g., Korsmeyer–Peppas). These observations support the plausibility and external validity of our findings for Labrafac/petrolatum semisolid systems.

However, the correlation with ex vivo permeation was not linear: LSV, despite releasing less drug, promoted a higher permeation (Figure 6). The receptor medium composed of PBS–Transcutol (50:50 *v*/*v*) was selected to ensure sink conditions during the ex vivo skin permeation studies, since the aqueous solubility of baricitinib in PBS alone is very low. As previously demonstrated by our group, the inclusion of Transcutol markedly increases baricitinib solubility [25]. Additionally, the medium showed to be biocompatible with the skin, as confirmed by TEWL measurements at the end of the tests. Regarding the experimental conditions, a stirring speed of 600 rpm was used to minimize the stagnant boundary layer beneath the membrane and to better emulate the in vivo situation, where continuous blood flow removes the permeated drug from the dermal–vascular interface. This stirring rate has been recommended by methodological guidelines [50] and is frequently applied in Franz diffusion studies to maintain homogeneous receptor conditions and reproducible flux measurements [51]. This finding shows that occlusivity and the vehicle’s ability to alter the skin barrier are as determining as the release rate in the final absorption profile [52,53]. Percutaneous absorption is primarily a passive diffusion process and can be described by Fick’s laws. However, the true driving force for transdermal transport is the thermodynamic activity—that is, the chemical-potential gradient of the drug between the formulation and the skin—rather than the nominal concentration alone. When the drug is near or at saturation in the vehicle, its thermodynamic activity is maximized and the potential for partitioning into the stratum corneum increases. Vehicle occlusivity modulates this process through two principal mechanisms. First, occlusive excipients (e.g., petrolatum, mineral oil, dimethicone) reduce transepidermal water loss (TEWL) and thereby increase stratum corneum hydration, which typically raises the diffusion coefficient within the barrier and can enhance permeation for lipophilic compounds. Second, occlusion and vehicle composition influence solvent evaporation: volatile components will evaporate from non-occlusive vehicles, concentrating the drug and potentially increasing its thermodynamic activity at the skin surface, whereas highly occlusive vehicles maintain solvent and drug in a solubilized state, which can lower thermodynamic activity and the net driving force. Thus, the net effect of occlusivity on permeation depends on the balance between increased skin hydration (which tends to increase permeability) and changes in the drug’s thermodynamic activity (which may increase or decrease the diffusion gradient). In our formulations, the relative proportions of Labrafac and liquid versus solid vaseline modulate occlusivity and co-solvent behavior, providing a mechanistic explanation for the differences in release and permeation observed in the permeation test [54].

A relevant aspect was differential cutaneous retention: LLV presented the highest amount of BCT accumulated in the skin, followed by lipid control (L) and finally LSV. These results suggest that the lower viscosity of LLV favors intracutaneous deposition and accumulation of the drug in the epidermal matrix, which is desirable for topical therapies targeting psoriasis lesions, where a sustained local action rather than systemic penetration is sought [55]. This balance between retention and permeation is crucial for optimizing the safety of topical BCT formulations, given the cardiovascular and thrombotic risks associated with their oral use [13,14].

No formulation induced irritation in the HET-CAM assay or in vivo models, consistent with the non-irritating profile of excipients such as MCT and petrolatum. Furthermore, in humans, excipients (placebo formulations) have been shown to transiently improve stratum corneum hydration and reduce transepidermal water loss, confirming an emollient-occlusive effect. The observed changes in SCH and TEWL may be related to the occlusive capacity of the formulations and their ability to reinforce the epidermal barrier. The water–Transcutol solution, used as a non-occlusive control (Supplementary Material), induced only an initial increase in the TEWL values. This behavior is consistent with the volatility of the vehicle and the penetration-enhancing properties of Transcutol, which may even transiently disrupt stratum corneum lipid organization, thereby preventing sustained hydration. Labrafac (L), composed of medium-chain triglycerides, exhibited an increase in SCH accompanied by a slight reduction in TEWL, indicating a weak semi-occlusive effect. Medium-chain triglycerides act primarily as emollients, improving skin softness and flexibility, but they do not form a continuous film capable of effectively limiting transepidermal water evaporation. The incorporation of liquid petrolatum into Labrafac (LLV) resulted in a more pronounced reduction in TEWL and a concomitant increase in SCH. Liquid petrolatum enhances film continuity and viscosity at the skin surface, leading to a moderate occlusive effect and improved water retention within the stratum corneum. Similar effects were observed for the formulation containing petrolatum in its semi-solid form (LSV). Petrolatum is known to form a continuous, semi-solid lipid film that markedly limits water evaporation. Accordingly, LSV exhibited TEWL reduction and the progressive SCH increase. Therefore, these findings are consistent with the literature, which attributes a dual function to lipid formulations: serving as both a drug vehicle and a skin barrier restorer [56]. Furthermore, the use of oily substances could make the skin softer by filling in the gaps between the partially loose skin scales (as occurs in psoriasis). This can restore the ability of the intercellular lipid bilayers to absorb, retain, and distribute water [57]. Although excipients such as Vaseline and MCT have a well-documented history of safe cosmetic use, the overall formulation and vehicle composition can significantly influence cutaneous responses. The combination of ingredients may modify key parameters such as skin penetration, occlusion, and the release kinetics of other actives. Therefore, even established excipients may behave differently when incorporated into a new formulation matrix [58,59]. We are fully aware that the use of healthy volunteers represents a limitation of the study, as psoriasis is characterized by an impaired skin barrier, chronic inflammation, and increased sensitivity. Therefore, results obtained on healthy skin cannot fully predict tolerance in psoriatic skin. Nevertheless, as a preliminary step, it is ethically and scientifically sound to first assess the irritant potential of a novel formulation in healthy individuals. This approach minimizes the risk of exposing patients with compromised skin to potentially irritating formulations, allows early detection of significant irritant reactions, and facilitates optimization of the formulation before conducting studies in more vulnerable populations.

Both formulations showed anti-inflammatory efficacy in the murine model of imiquimod-induced psoriasis, reducing erythema, epidermal thickening and loss of barrier function. Histology confirmed the attenuation of hyperplasia and vascular dilation, effects that reflect the inhibition of the JAK-STAT cascade and possibly the decrease of cytokines such as IL-6 and IL-23, mechanisms previously described for BCT [9], which is why this result validates that, even with different release and retention profiles, both formulations allow reaching sufficient tissue concentrations to modulate the local inflammatory response. The histological findings confirm that imiquimod induced epidermal hyperplasia and structural disorganization in dorsal skin, while both formulations (LSV and LLV) effectively maintained normal epidermal architecture. In ear skin, however, the inflammatory response appeared to be primarily localized within the dermis, as indicated by the presence of dilated and blood-filled vessels, rather than affecting the epidermal compartment. This explains why no significant differences in epidermal thickness were detected among ear samples, despite clear dermal inflammation in the positive control group. Similar dermis-restricted inflammatory patterns have been described in murine models of topical imiquimod-induced irritation [60].

An important point of discussion is the inverse relationship between permeation and retention observed between LSV and LLV; while LSV maximized transcutaneous permeation, LLV optimized cutaneous retention. This phenomenon can be interpreted within the framework of the vehicle-stratum corneum partition coefficient and the microstructure of the excipients. Solid petrolatum, being more occlusive, probably favored hydration of the stratum corneum and the opening of transient diffusive channels, facilitating passage into the dermis and circulation, while liquid petrolatum, less occlusive, retained the drug in the epidermis, acting as a reservoir [46]. Therefore, this duality can be exploited in therapeutic customization: an LLV-type formulation would be ideal for localized lesions that require prolonged action on site.

This study has several limitations that should be acknowledged. The sample size in both animal and human studies was relatively small, which may limit the statistical power and generalizability of the findings. In addition, the relatively high standard deviation observed in some in vitro release and ex vivo permeation data can be partly attributed to the limited number of replicates (*n* = 6), which increases the influence of inter-sample variability. The ex vivo human skin experiments were intended to provide preliminary insight into the ability of the formulations to deliver baricitinib (BCT) into the skin. Although direct comparison with previously published data is limited due to the novelty of topical BCT application, the drug levels retained in the skin were considered potentially effective based on the positive in vivo outcomes in mice. However, given that murine skin is more permeable than human skin, extrapolation between models should be interpreted with caution.

Moreover, although the imiquimod-induced psoriasis model is widely accepted for evaluating psoriatic inflammation, the number of animals per group (*n* = 5) was determined according to ethical principles of reduction and institutional guidelines, aiming to minimize animal use in exploratory efficacy and tolerability studies. This sample size allows the detection of relatively large treatment effects but provides limited power to identify smaller differences; therefore, confirmatory studies with larger group sizes are warranted to strengthen the robustness and reproducibility of the findings. Additionally, a limitation of the exploratory in vivo efficacy study is the absence of a placebo control group, this decision was aligned with the go/no-go nature of the study, whose primary objective was to determine whether the formulations exhibited sufficient biological activity to warrant further development. The IMQ group provided a robust and reproducible negative control, allowing sensitive detection of therapeutic effects while keeping the design efficient at this early stage. Both formulations reduced erythema, scaling, and skin thickening compared with IMQ controls, meeting the predefined “go” criteria. These results support progression to a pivotal preclinical efficacy study, in which a full placebo arm will be included to provide rigorous and confirmatory comparative data.

Finally, long-term stability testing and sensory or user acceptability evaluations were not included in this work and should be addressed in future studies. Despite these limitations, the present results offer valuable preliminary evidence supporting the topical potential of baricitinib formulations and justify further preclinical and clinical investigations.

This study demonstrates that the choice of petrolatum type has a decisive impact on the properties of topically applied BCT. The results suggest that lipid formulations with liquid petrolatum offer a more suitable profile for topical therapies in psoriasis, favoring local action. This information provides important supporting evidence for the rational design of topical formulations of JAK inhibitors, offering alternatives that could overcome the limitations of oral administration.

## 5. Conclusions

This study demonstrated that vehicle occlusivity, determined by the type of petrolatum, modulates the biopharmaceutical performance and skin delivery profile of baricitinib. Both MCT-based formulations (LLV and LSV) exhibited adequate physicochemical properties. The use of liquid petrolatum formulation (LLV) resulted in a Newtonian flow, greater spreadability, and maximized drug retention within the skin, suggesting a prolonged local action.

Furthermore, the formulations also showed good biocompatibility for dermal application, were non-irritating in the safety assessments and improved skin hydration in human volunteers, and displayed anti-psoriatic efficacy in the exploratory imiquimod-induced murine model, with histological analysis revealing epidermal and dermal structures comparable to healthy tissues, suggesting that they are potential candidates for psoriasis treatment.

Overall, these findings confirm that excipient selection is a significant factor in the rational design of effective topical JAK inhibitor formulations, offering distinct therapeutic profiles tailored to desired local retention and enhanced permeation.

## Figures and Tables

**Table 1:** Experimental formulation matrix.

Formulations	Denomination	Factors	
		(A)	(B)
F1	LSV_1_	+	+
F2	LSV_2_	+	−
F3	LLV_1_	−	+
F4	LLV_2_	−	−

**Table 2:** Composition of formulations with BCT.

Components	% *w*/*w*				
	LSV_1_	LSV_2_	LLV_1_	LLV_2_	L
BCT	0.04	0.04	0.04	0.04	0.04
Medium-chain triglyceride	39.96	69.96	39.96	69.96	99.96
Solid petroleum jelly	60.00	30.00	NA	NA	NA
Liquid petroleum jelly	NA	NA	60.00	30.00	NA

**Table 3:** HET-CAM test scoring system [35].

Time	Hyperemia	Hemorrhage	Coagulation/Opacity
<30 s	5	7	9
31–119 s	3	5	7
120–299 s	1	3	5
>300 s (no effect)	0	0	0

**Table 4:** Physical and chemical characterization of BCT formulations stored at 30 ± 2 °C/65 ± 5% RH.

Formulation	Time	pH ± SD	%Assay ± SD	Appearance ^a^	Color	Phase Separation
LSV1	1 day	5.9 ± 0.1	99.9 ± 0.1	Homogeneous, semi-solid	Pearly white	Absent
	30 days	5.0 ± 0.1	85.1 ± 0.3	Light precipitation visible	Opaque white	Present
	60 days	*	*	Precipitation	Opaque withe	Present
LSV2	1 day	5.8 ± 0.1	99.9 ± 0.1	Homogeneous, semi-solid	Pearly white	Absent
	30 days	5.7 ± 0.1	98.8 ± 0.2	Homogeneous, semi-solid	Pearly white	Absent
	60 days	5.6 ± 0.1	97.1 ± 0.2	Homogeneous, semi-solid	Pearly white	Absent
LLV1	1 day	6.0 ± 0.1	99.9 ± 0.1	Homogeneous, fluid	Slightly yellowish	Absent
	30 days	5.1 ± 0.1	82.0 ± 0.2	Slight turbidity	Yellowish	Present
	60 days	*	*	Precipitation	Yellowish	Present
LLV2	1 day	5.9 ± 0.1	100.0 ± 0.1	Homogeneous, fluid	Slightly yellowish	Absent
	30 days	5.8 ± 0.1	98.1 ± 0.1	Homogeneous, fluid	Slightly yellowish	Absent
	60 days	5.7 ± 0.1	96.9 ± 0.2	Homogeneous, fluid	Slightly yellowish	Absent

* Excluded due to precipitation observed. ^a^ Visual inspection included evaluation of the color, homogeneity, and phase separation.

**Table 5:** Physical and chemical characterization of BCT formulations stored at 40 ± 2 °C/75 ± 5% RH.

Formulation	Time	pH ± SD	%Assay ± SD	Appearance	Color	Phase Separation
LSV1	Day 1	5.9 ± 0.1	99.9 ± 0.1	Homogeneous, semisolid	Pearly white	Absent
	Day 30	*	*	Slight turbidity, light precipitation visible	Opaque white	Present
	Day 60	*	*	Precipitation	Opaque white	Present
LSV2	Day 1	5.8 ± 0.1	99.9 ± 0.1	Homogeneous, semisolid	Pearly white	Absent
	Day 30	5.6 ± 0.1	97.3 ± 0.3	Homogeneous, semisolid	Pearly white	Absent
	Day 60	5.5 ± 0.1	96.1 ± 0.3	Homogeneous, semisolid	Pearly white	Absent
LLV1	Day 1	6.0 ± 0.1	99.9 ± 0.1	Homogeneous, fluid	Slightly yellowish	Absent
	Day 30	*	*	Turbidity, visible precipitate	Opaque yellow	Present
	Day 60	*	*	Precipitation	Opaque yellow	Present
LLV2	Day 1	5.9 ± 0.1	100.0 ± 0.1	Homogeneous, fluid	Slightly yellowish	Absent
	Day 30	5.7 ± 0.1	96.1 ± 0.2	Homogeneous, fluid	Slightly yellowish	Absent
	Day 60	5.4 ± 0.2	95.7 ± 0.3	Homogeneous, fluid	Slightly yellowish	Absent

* Excluded due to precipitation observed.

**Table 6:** Degradation kinetics. K: degradation rate constant; r^2^: determination coefficients and t_90_: time at which 90% of the initial drug content remains. * Statistically different (*p* < 0.001).

Formulation	Temperature (°C)	k	r^2^	t_90_ (Days)
LSV2	30	0.06580 *	0.946	151.98
LSV2	40	0.10700 *	0.992	93.46
LLV2	30	0.08062 *	0.984	124.03
LLV2	40	0.13303 *	1.000	75.17

**Table 7:** Rheological behaviour and mathematical model fitting of the formulations tested. The goodness of fit was based on the r value, and the mathematical model selected is highlighted in grey. Results are expressed as the mean ± SD of 3 replicates.

Sample	Temperature	Viscosity at 100 s^−1^ (mPa·s)	Rheological Behaviour and Model			
			Stretch Ramp up	r	Stretch Ramp down	r
L	25 °C	9.16 ± 0.01	Newton	1	Newton	1
			Bingham	1	Bingham	1
			Ostwald de Waele	1	Ostwald de Waele	1
			Herschel–Bulkley	1	Herschel–Bulkley	1
			Casson	0.9999	Casson	0.9999
			Cross	0.9983	Cross	0.9943
LLV	25 °C	10.62 ± 0.01	Newton	1	Newton	1
			Bingham	1	Bingham	1
			Ostwald de Waele	1	Ostwald de Waele	1
			Herschel–Bulkley	1	Herschel–Bulkley	1
			Casson	1	Casson	1
			Cross	0.9947	Cross	0.995
LSV	25 °C	17.88 ± 0.09	Newton	0.9899	Cross	0.9954
			Bingham	0.9989	Bingham	0.9999
			Ostwald de Waele	0.9998	Ostwald de Waele	0.9994
			Herschel–Bulkley	1	Herschel–Bulkley	1
			Casson	0.9999	Casson	0.9998
			Cross	1	Cross	1

**Table 8:** Rheological parameters of L, LLV, and LSV formulations obtained from the best-fitting rheological model.

Formulation	Model	η (Pa·s)	η_0_ (Pa·s)	η∞ (Pa·s)	K (s^−1^)	n	R^2^
L	Newton	0.0092	–	–	–	–	1.000
LLV	Newton	0.0106	–	–	–	–	1.000
LSV	Cross	–	0.1771	0.0133	0.1730	0.5512	1.000

η: constant viscosity; η_0_: zero-shear viscosity; η∞: infinite-shear viscosity; K: characteristic shear rate; n: flow behavior index describing the degree of pseudoplasticity; and R^2^ indicates the goodness of fit for each model.

**Table 9:** Goodness-of-fit of the kinetic models evaluated, and the Diffusion exponent to evaluate the diffusion mechanism.

Formulation	Zero Order	First-Order	Second Order	Higuchi	Korsmeyer–Peppas	Diffusion Exponent (n)
LLV	0.9306	0.9305	0.9460	0.8375	0.9076	0.7836 ± 0.1174
LSV	0.9307	0.9370	0.9357	0.8874	0.9362	0.7348 ± 0.0942
L	0.8870	0.8870	0.9054	0.8118	0.8736	0.7612 ± 0.1379

**Table 10:** Transepidermal water loss (TEWL, g/h/m^2^) values measured before (at the beginning) and after (at the end) the permeation experiments for each formulation.

Skin Disc	L		LLV		LSV	
Replicate	At the Beginning	At the End	At the Beginning	At the End	At the Beginning	At the End
1	8.75	9.02	8.12	8.40	9.66	9.90
2	9.90	9.96	9.73	9.90	8.42	8.59
3	9.46	9.80	9.20	9.38	8.36	8.49
4	9.20	9.36	9.42	9.73	8.37	8.85
5	8.31	8.53	8.04	8.38	8.61	9.10
6	8.31	8.56	9.94	9.90	9.05	9.47

**Table 11:** Biopharmaceutical parameters of BCT formulations through ex vivo human skin. Values represent mean ± SD (*n* = 6).

Formulation	*Qp*_5_ (µg)	*Qp*_24_ (µg)	*Q_ret_* (µg/ g skin/cm^2^)
LSV	1.58 ± 0.10	2.08 ± 0.14	49.72 ± 8.95
LLV	1.10 ± 0.10	2.85 ± 0.11	94.05 ± 17.87
L	0.75 ± 0.09	0.96 ± 0.10	84.18 ± 15.15

*Qp*_5_ = amount of drug permeated through the skin at 5 h; *Qp*_24_ = amount of drug permeated at 24 h; and *Qret* = amount of drug retained in the skin.

**Table 12:** Summary of the quantitative histomorphometric analysis of viable epidermal thickness (µm) in dorsal and ear skin samples from the imiquimod-induced psoriasis model. Data are expressed as mean ± SD of 10 measurements for each image. Statistical significance was determined by one-way ANOVA followed by Tukey’s post hoc test.

Anatomical Site	Control	LSV	LLV	*p*-Value	Result
Dorsal	89.2 ± 12.1	77.5 ± 6.4	75.3 ± 5.1	0.024	LSV and LLV significantly reduce epidermal thickness compared to the positive control
Ear	39.1 ± 6.3	37.0 ± 5.7	37.8 ± 5.0	0.81	No significant statistical differences

## Data Availability

The data presented in this study are available in this article.

## Supplementary Materials

The following supporting information can be downloaded at https://www.mdpi.com/article/10.3390/pharmaceutics18010008/s1. Figure S1: Comparison of the evolution of the biomechanical properties of skin tolerance in humans after the application of the formulations with regard to the basal values: (A) SCH of L, LLV, LSV and W/T sol, and (B) TEWL of L, LLV, LSV and W/T sol. Data expressed as the mean ± SD (*n* = 10).

## Abbreviations

The following abbreviations are used in this manuscript:

BCT	Baricitinib
RH	Relative Humidity
L	Formulation with Labrafac^®^ Lipohile
LSV	Formulation with solid petroleum jelly
LLV	Formulation with liquid petroleum jelly
MCT	Medium-chain tryglicerides
HET-CAM	Hen’s Egg Test-Chorioallantoic Membrane
IMQ	Imiquimod
TEWL	Transepidermal water loss
SCH	Stratum corneum hydration
W/T sol	Solution composed of water-Transcutol (1:1, *v*/*v*)
