# Supplementary material for: The Impact of Vehicle Occlusivity on Skin Delivery and Activity of a Janus Kinase Inhibitor: Comparison of Oil-Based Formulations
Source: Pharmaceutics. 2025 Dec 20;18(1):8. doi: 10.3390/pharmaceutics18010008 (PMC12844953; doi:10.3390/pharmaceutics18010008)
Supplement: Supplementary file 1 [file pharmaceutics-18-00008-s001.zip › pharmaceutics-3942983-supplementary.pdf]

pharmaceutics

MDPI

Supplementary Material

The impact of vehicle occlusivity on skin delivery and activity of a janus kinase inhibitor: comparison of oil-based formulations

Paulo Sarango-Granda [1,3,‡], Roya Mohammadi-Meyabadi [2,3,‡], Antonio Braza [2], Lilian Sosa [4,5], Joaquim Suñer-Carbó [2,3], Mireia Mallandrich [2,3,*] and Ana Cristina Calpena [2,3,*]

[1] Departamento de Química, Facultad de Ciencias Exactas y Naturales, Universidad Técnica Particular de Loja (UTPL), Paris SN y Praga, 1101607 Loja, Ecuador; pcsarango@utpl.edu.ec (P.S-G).
[2] Departament de Farmàcia i Tecnologia Farmacèutica, i Fisicoquímica, Facultat de Farmàcia i Ciències de l'Alimentació, Universitat de Barcelona (UB), Av. Joan XXIII, 27-31, 08028 Barcelona, España; anacalpena@ub.edu; rmohammo31@alumnes.ub.edu; mireia.mallandrich@ub.edu; jsunerc@cofgi.org; braza@ub.edu (A.C., R.M-M., M.M., J.S-C., A.B.).
[3] Institut de Nanociència i Nanotecnologia, Universitat de Barcelona (UB), Av. Diagonal 645, 08028 Barcelona, España;
[4] Instituto de Investigaciones en Microbiología (IIM), Facultad de Ciencias, Universidad Nacional Autónoma de Honduras (UNAH), Tegucigalpa 11101, Honduras; lilian.sosa@unah.edu.hn (L.S).
[5] Centro Experimental en Biociencia (CENBIO), Facultad de Ciencias Químicas y Farmacia, Universidad Nacional Autónoma de Honduras (UNAH), Tegucigalpa 11101, Honduras.
‡ Equally contributed
* Correspondence: anacalpena@ub.edu; mireia.mallandrich@ub.edu

Comparison of the SCH and TEWL between occlusive and non-occlusive vehicles on the skin of healthy volunteers

Figure S1 shows the comparison of SCH and TEWL between the MCT-based formulations which have different degrees of occlusive effect because of the medium chain triglycerides, and the liquid and solid petrolatum and a non-occlusive vehicle such as an aqueous solution (W/T sol) composed of water and transcutol (1:1, *v/v*).

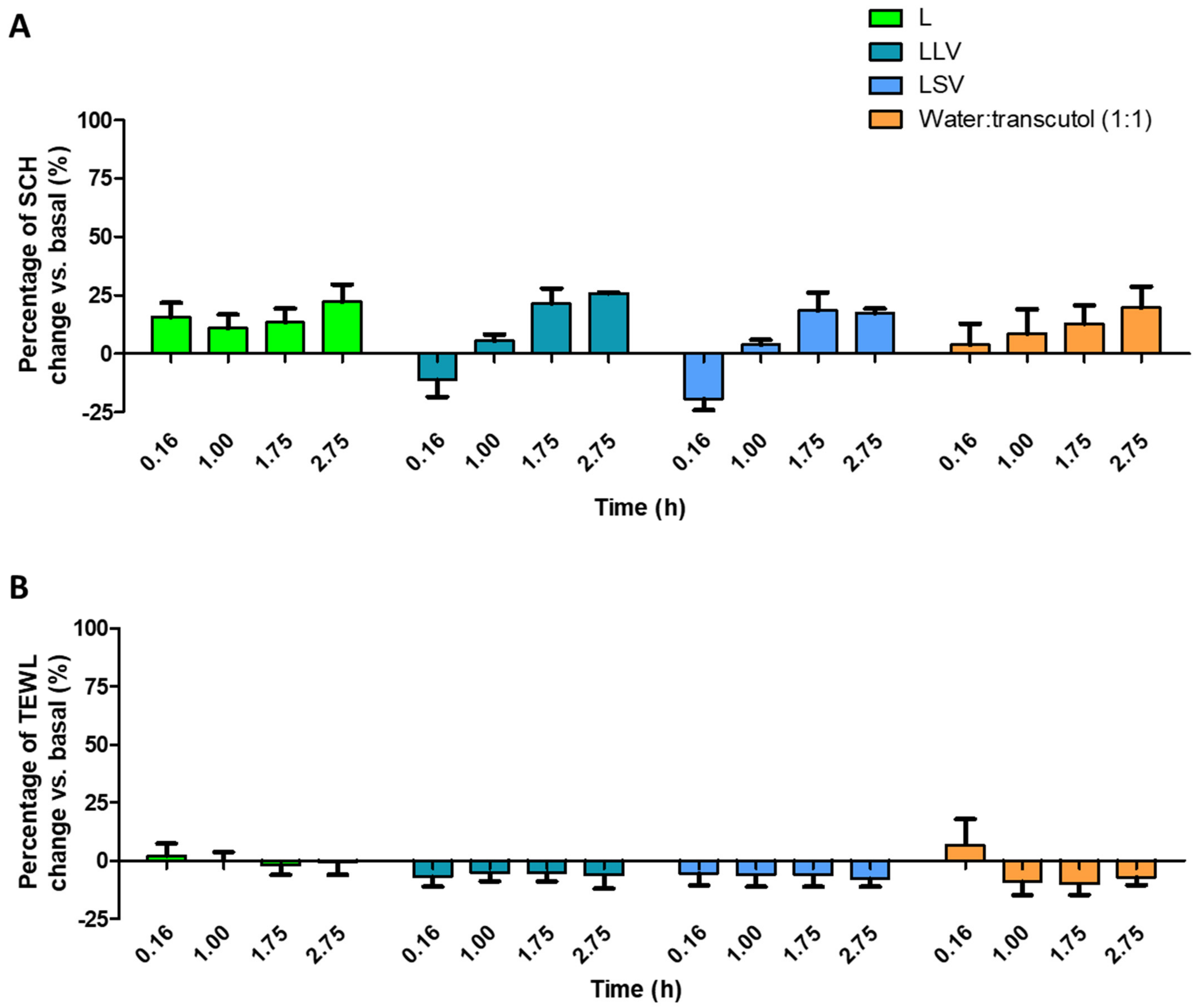

Figure S1. Comparison of the evolution of the biomechanical properties of the skin tolerance in humans after the application of the formulations with regard to the basal values: (**A**) SCH of L, LLV, LSV and W/T sol; and (**B**) TEWL of L, LLV, LSV and W/T sol. Data expressed as mean ± SD (*n* = *10*).
